# A Case of Airway Obstruction Secondary to Acute Haemorrhage into a Benign Thyroid Cyst

**DOI:** 10.1155/2014/372369

**Published:** 2014-08-21

**Authors:** Ravi Vijapurapu, Kamal Kaur, Neil H. Crooks

**Affiliations:** ^1^Sandwell & West Birmingham Hospitals NHS Trust, Dudley Road, Birmingham, B18 7QH, UK; ^2^Academic Department of Anaesthesia, Critical Care, Pain & Resuscitation, Birmingham Heartlands Hospital, 1st Floor MIDRU Building, Bordesley Green East, Birmingham B9 5SS, UK

## Abstract

A 70-year-old female, with a history of progressive dyspnoea, was admitted to the critical care unit after successful resuscitation following a witnessed, out of hospital cardiorespiratory arrest. A presumptive diagnosis of cardiorespiratory arrest secondary to an exacerbation of chronic obstructive pulmonary disease was made. However, on more detailed examination a large anterior, midline neck mass was noted. Following tracheal intubation, a computerised tomography scan of the patient's neck and thorax revealed a seven-centimetre, well-defined, nonenhancing, rounded homogeneous opacity at the thoracic inlet, consistent with a large midline thyroid cyst. Needle aspiration of the cyst was performed and yielded approximately 50 mL of frank blood. After an uncomplicated tracheal extubation and recovery, an elective subtotal thyroidectomy was performed prior to hospital discharge. Histology of the specimen revealed a benign thyroid cyst within a multinodular goitre. Euthyroid multinodular goitres are more likely to be managed conservatively due to an asymptomatic clinical course in most patients. However, the risk of respiratory distress and acute airway obstruction from tracheal compression or acute haemorrhage should be kept in mind. Patients at risk of this life threatening complication should be managed with elective thyroidectomy to reduce morbidity and mortality.

## 1. Introduction

Thyroid haemorrhages are usually small and tend to cause relatively minor symptoms such as pain and local discomfort. However, it has been reported that significant haemorrhage into a thyroid cyst or nodule can lead to a rapidly expanding haematoma with subsequent airway compromise. These haemorrhagic events commonly occur after exertion or trauma (such as fine needle aspiration) [1]. We present a case of acute airway obstruction and subsequent cardiac arrest, which was initially diagnosed as a severe exacerbation of chronic obstructive pulmonary disease (COPD) but on further evaluation was found to be the result of spontaneous haemorrhage into a preexisting thyroid cyst.

## 2. Case Description

A 70-year-old female was admitted to a local district general hospital following an out of hospital cardiorespiratory arrest. The patient had called an ambulance herself due to difficulty in breathing. On arrival, paramedics found a patient with acute dyspnoea and stridor who collapsed shortly afterwards with loss of cardiac output. Cardiopulmonary resuscitation (CPR) was commenced and there was return of spontaneous circulation after three minutes. The cardiac rhythm during these events was not recorded and no resuscitation drugs were administered. The patient was transferred to the emergency department where, due to continued poor respiratory effort, the patient's trachea was intubated after a rapid sequence induction and she was transferred to the critical care unit (CCU) for ongoing management.

### 2.1. Clinical Course

A presumptive diagnosis of cardiorespiratory arrest secondary to an exacerbation of COPD was initially made. A collateral history taken from relatives revealed that she had become progressively more breathless over the preceding three weeks, and despite a course of oral antibiotics and steroids there was little improvement. More detailed investigation of the patient's past medical history revealed that she was known to have a cystic swelling in the anterior cervical area. This had increased in size over the previous three weeks and was associated with progressive dysphagia and dyspnoea. The patient was awaiting outpatient otorhinolaryngology review.

**Figure 1:**
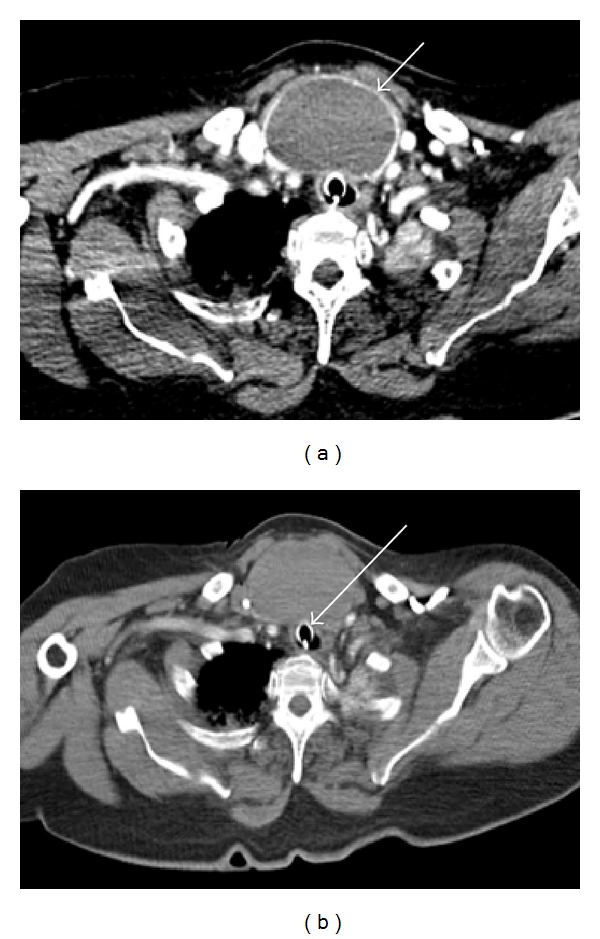
Axial section CT imaging of the neck showing a 7 cm anterior cervical mass. A well-defined, nonenhancing, rounded homogenous opacity at the thoracic inlet, within the thyroid isthmus. The endotracheal tube can be visualised within the compressed trachea in (b).

**Figure 2:**
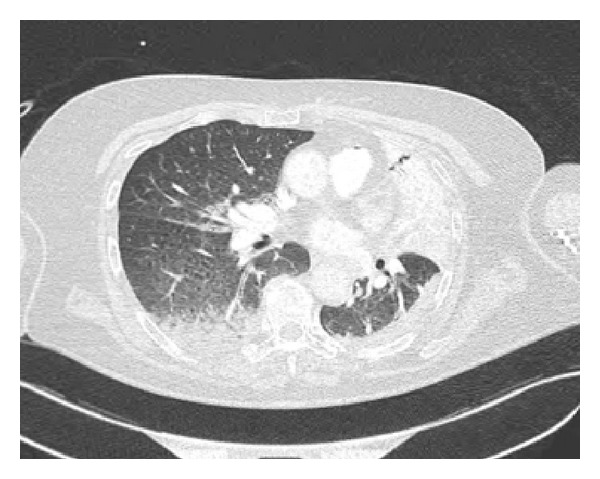
Axial section CT image of the thorax. Bilateral consolidative change with air bronchogram, more extensive within the left lung.

In view of this history, computerised tomography (CT) imaging of the neck and thorax was performed. This revealed a seven-centimetre, well-defined, nonenhancing, rounded homogenous opacity at the thoracic inlet, consistent with a large midline thyroid cyst (Figure 1). Significant consolidative change within the inferior lobe of the left lung was also noted (Figure 2). Subsequent clinical examination revealed a large cystic swelling occupying the whole of the thyroid gland. Needle aspiration of the cyst yielded approximately 50 mL of frank blood.

On review of the clinical history and taking into account these new findings, it was felt that the patient had suffered acute airway obstruction secondary to haemorrhage into a preexisting thyroid cyst.

The patient was invasively ventilated for total of seven days before successful extubation to noninvasive ventilation. She received antibiotic therapy during this time for community-acquired pneumonia. In view of the progressive thyroid swelling causing dysphagia and airway compromise, it was felt to be highly likely that the patient had been suffering from chronic aspiration over the preceding weeks. This, in combination with acute airway obstruction, had caused a significant deterioration in the patient's condition leading to cardiorespiratory arrest.

### 2.2. Outcome and Followup

Following discharge from CCU, clinical examination revealed that the thyroid cyst had reaccumulated. Consequently, an elective subtotal thyroidectomy was performed and a large retrosternal cyst within a multinodular goitre was excised. Histopathological examination of the specimen identified a benign cyst within the isthmus of the thyroid gland. There was no evidence of malignant change.

The patient was subsequently discharged from hospital 6 days following the operation and reviewed in outpatient clinic three weeks later. She was commenced on levothyroxine therapy and has remained clinically and biochemically euthyroid, with no further enlargement of the remnant thyroid tissue.

## 3. Discussion

Thyroid nodules and cysts are extremely common and usually benign, with only 5% being malignant [2]. Euthyroid multinodular goitres are more likely to be managed conservatively due to an asymptomatic clinical course in most patients. Surgery is reserved for those cases refractory to medical therapy. These types of goitres are usually slow-growing and indolent in nature, remaining relatively asymptomatic. However, the possibility of respiratory compromise due to tracheal compression should always be borne in mind when assessing these patients.

Acute haemorrhage within a goitre can lead to tracheal compression and acute airway obstruction. However, the literature suggests that haemorrhage into a thyroid cyst or nodule does not commonly cause significant symptoms and extensive haemorrhage is relatively uncommon [3, 4].

There are two proposed mechanisms that may lead to haemorrhage to a goitre, the first being the presence of abnormal thyroid vascular anatomy leading to weaker vessel walls and subsequent increased risk of rupture. In some cases, pathological specimens and real-time imaging have identified arteriovenous shunting into a cyst or nodule that causes diversion of blood under higher pressure into the cystic veins, with consequent extravasation within this structure [5]. Strenuous activity can lead to rupture of this aberrant vasculature and result in a thyroid haematoma.

The usual management for simple thyroid haematomata would be fine needle aspiration and drainage [6]. However in a case such as this, where a large volume thyroid cyst had filled entirely with blood clots, simple aspiration would not have been a suitable option alone. Although, successful extubation was possible after aspirating blood from the thyroid cyst and relieving the airway compression, the definitive management involved surgical decompression with thyroidectomy.

This case highlights that patients at risk of life-threatening complications of thyroid disease, such as tracheal compression and airway obstruction, should be managed with early surgical intervention in order to reduce morbidity and mortality. It also demonstrates the importance of thorough history-taking and clinical examination, particularly in a critical care setting, in order to avoid missing relevant clinical details.
